# Supplementary material for: POLQ-driven repair scars shape the immunogenic landscape of homologous recombination-deficient pancreatic cancer
Source: bioRxiv. 2026 Mar 17:2026.03.15.711961. Preprint. [Version 1] doi: 10.64898/2026.03.15.711961 (PMC13015423; doi:10.64898/2026.03.15.711961)
Supplement: Supplement 1 [file NIHPP2026.03.15.711961v1-supplement-1.pdf]

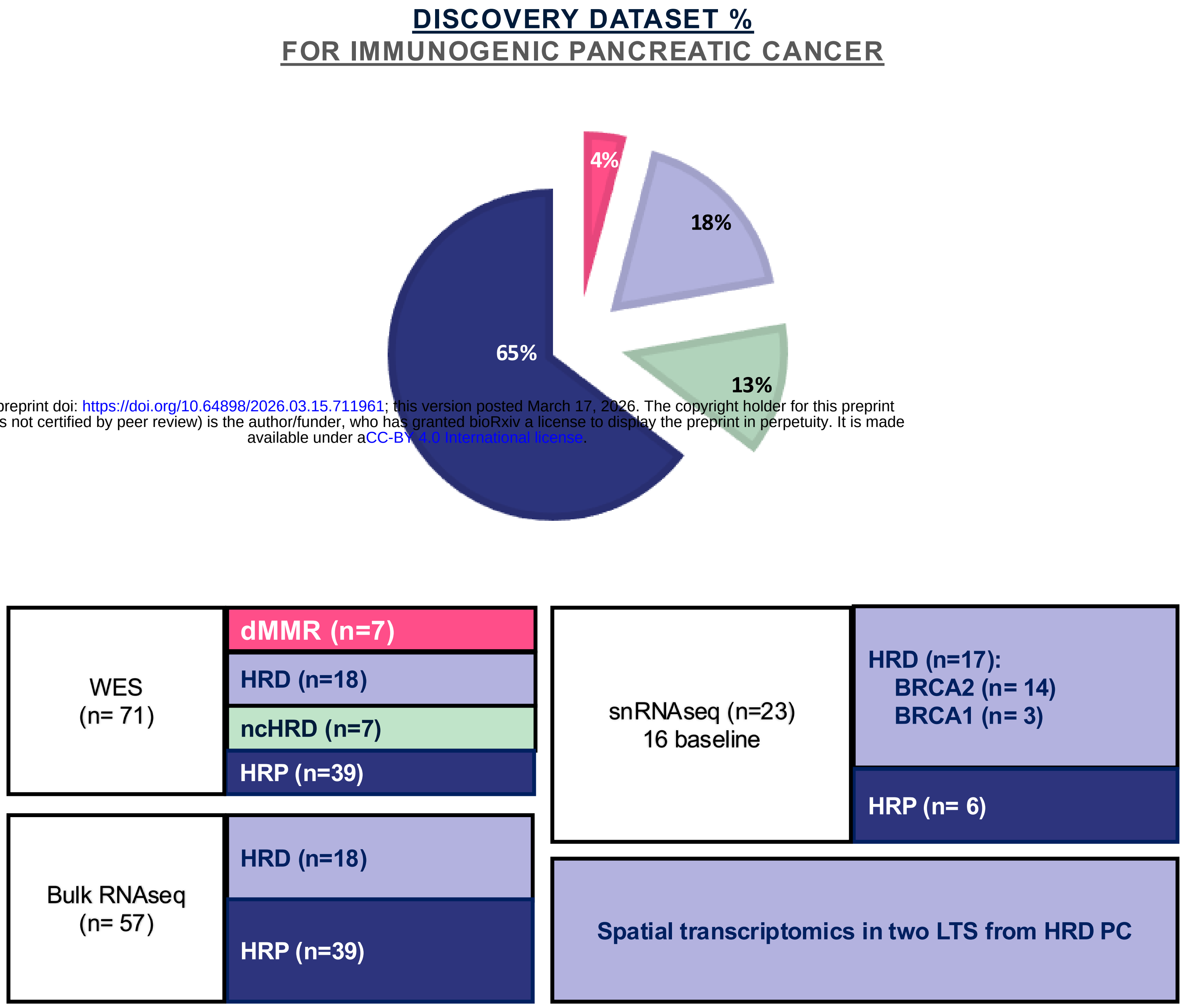

bioRxiv preprint doi: https://doi.org/10.64898/2026.03.15.711961; this version posted March 17, 2026. The copyright holder for this preprint (which was not certified by peer review) is the author/funder, who has granted bioRxiv a license to display the preprint in perpetuity. It is made available under aCC-BY 4.0 International license.

Figure S1. Multiomic profiling overview of the discovery immunogenic pancreatic cancer (iPC) dataset

Schematic summary of the iPC multiomic assay design, depicting data availability (WES, bulk RNA-seq, snRNA-seq, and mIHC/mIF) across DDR-defined subgroups (dMMR, HRD, ncHRD, HRP). Baseline and follow-up (FU) samples, including matched longitudinal sets (n = 5 pairs), are indicated.

Abbreviations: WES, whole exome sequencing; ecRNAseq, exome-captured RNAseq; snRNAseq, single-nucleus RNAseq; STS, short-term survivor; LTS, long-term survivor; mIHC, multiplxe immunohistochemistry; mIF, multiplex immunofluorescent; PC, pancreatic cancer; dMMR, mismatch repair deficient; HRD, homologous recombination deficient; ncHRD, non-core HRD; HRP, homologous recombination proficient

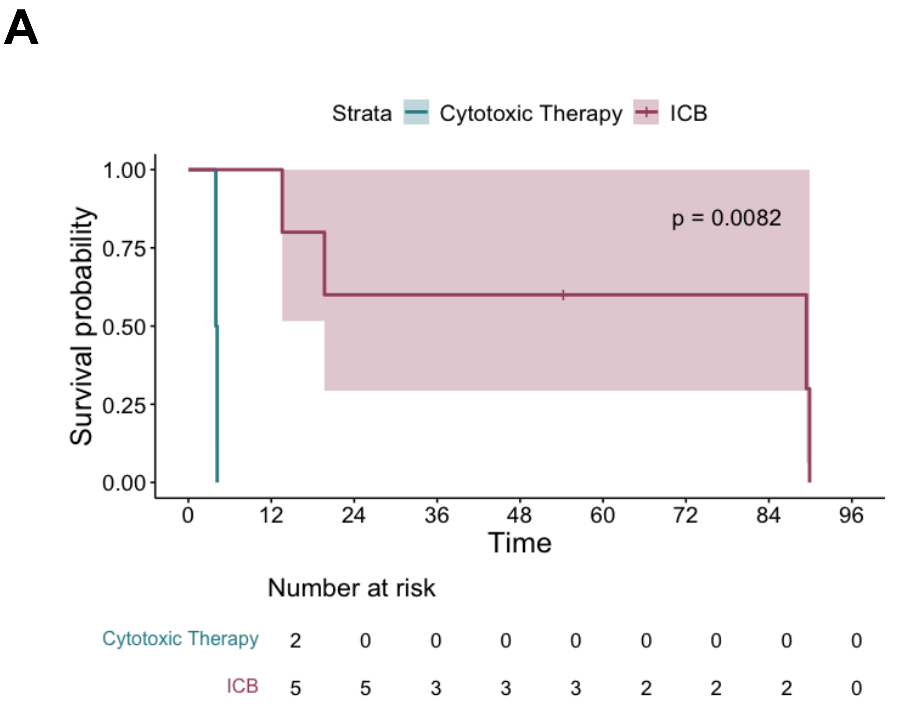

bioRxiv preprint doi: https://doi.org/10.64898/2026.03.15.711961; this version posted March 17, 2026. The copyright holder for this preprint (which was not certified by peer review) is the author/funder, who has granted bioRxiv a license to display the preprint in perpetuity. It is made available under aCC-BY 4.0 International license.

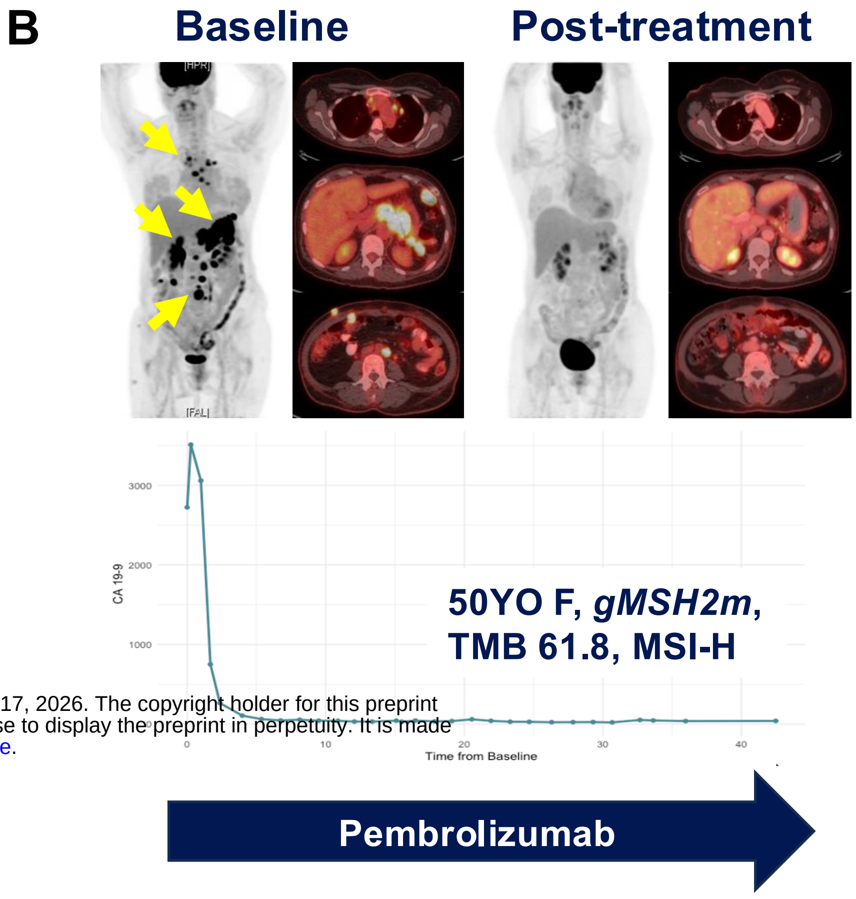

C

Circulating Immune Cells	Value (Reference)
**Absolute Lymphocytes**	▲ **4802 (49.5 %)** (850-3180 cells/mcl)
**CD8+ T**	▲ **1152** (209-768 cells/mcl lymph)
**CD4+T**	▲ **2257** (25-133 cells/mcl lymph)
CD4+45RA+ **naïve T**	▲ **1056** (89-789 cells/mcl lymph)
CD3+56+16+ **NKT**	▲ **1056** (19-199 cells/mcl)
CD3-19+ **B**	▲ **480** (85-441 cells/ mcl)

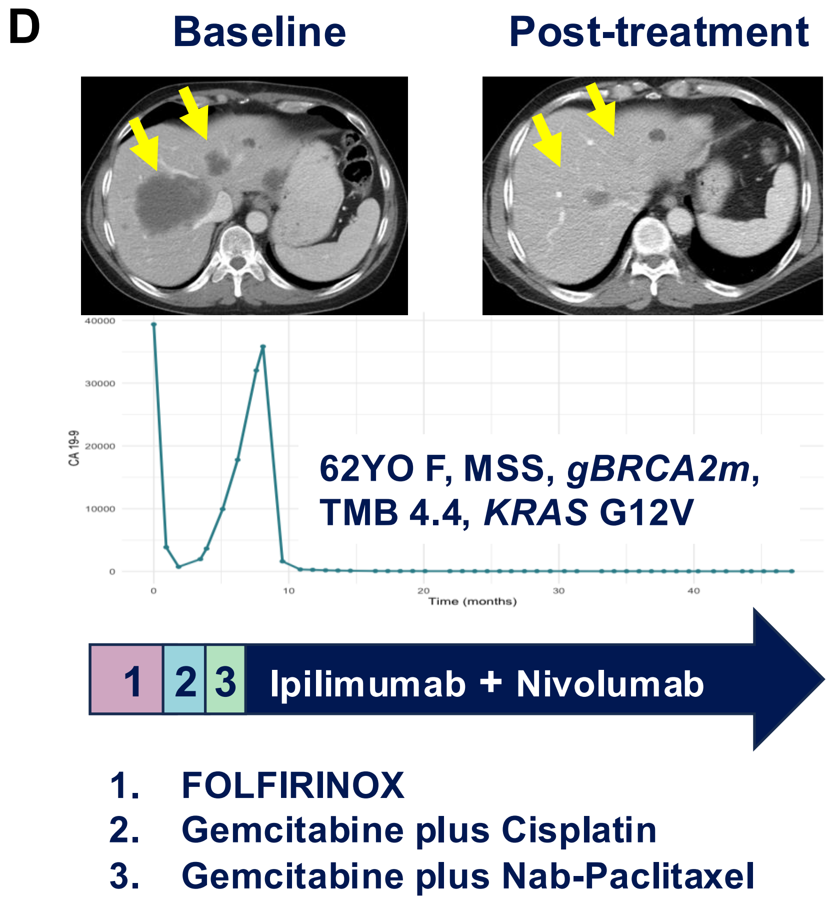

Figure S2. Clinical evidence of immunogenic pancreatic cancer with DNA damage repair deficiency

Mismatch repair deficient (dMMR) pancreatic cancer represents the most clearly immunogenic subset of pancreatic cancer responsive to immune checkpoint blockade (ICB). **(A)** Kaplan–Meier overall survival (OS) curve comparing patients with advanced dMMR pancreatic cancer treated with immune checkpoint blockade (ICB, red) versus cytotoxic chemotherapy (blue) (p=0.0082, N=7). **(B)** PET–CT imaging of an exceptional responder with metastatic pancreatic cancer harboring *gMSH2* mutation, MSI-H status, and high tumor mutational burden (TMB 61.8). Imaging demonstrates complete radiographic response following pembrolizumab therapy. Longitudinal CA19-9 levels show rapid normalization and sustained response during treatment. **(C)** Post-treatment peripheral immune profiling from the same patient demonstrating elevated circulating lymphocytes with increased $CD8^+$ T cells, $CD4^+$ T cells, and $CD3^+CD56^+CD16^+$ natural killer T (NKT) cells. **(D)** Radiographic response in a patient with metastatic pancreatic cancer harboring germline *BRCA2* mutation (TMB 4.4, MSS) treated with ipilimumab plus nivolumab in the fourth-line setting, illustrating potential immune responsiveness in homologous recombination deficient (HRD) pancreatic cancer.

bioRxiv preprint doi: https://doi.org/10.64898/2026.03.15.711961; this version posted March 17, 2026. The copyright holder for this preprint (which was not certified by peer review) is the author/funder, who has granted bioRxiv a license to display the preprint in perpetuity. It is made available under aCC-BY 4.0 International license.

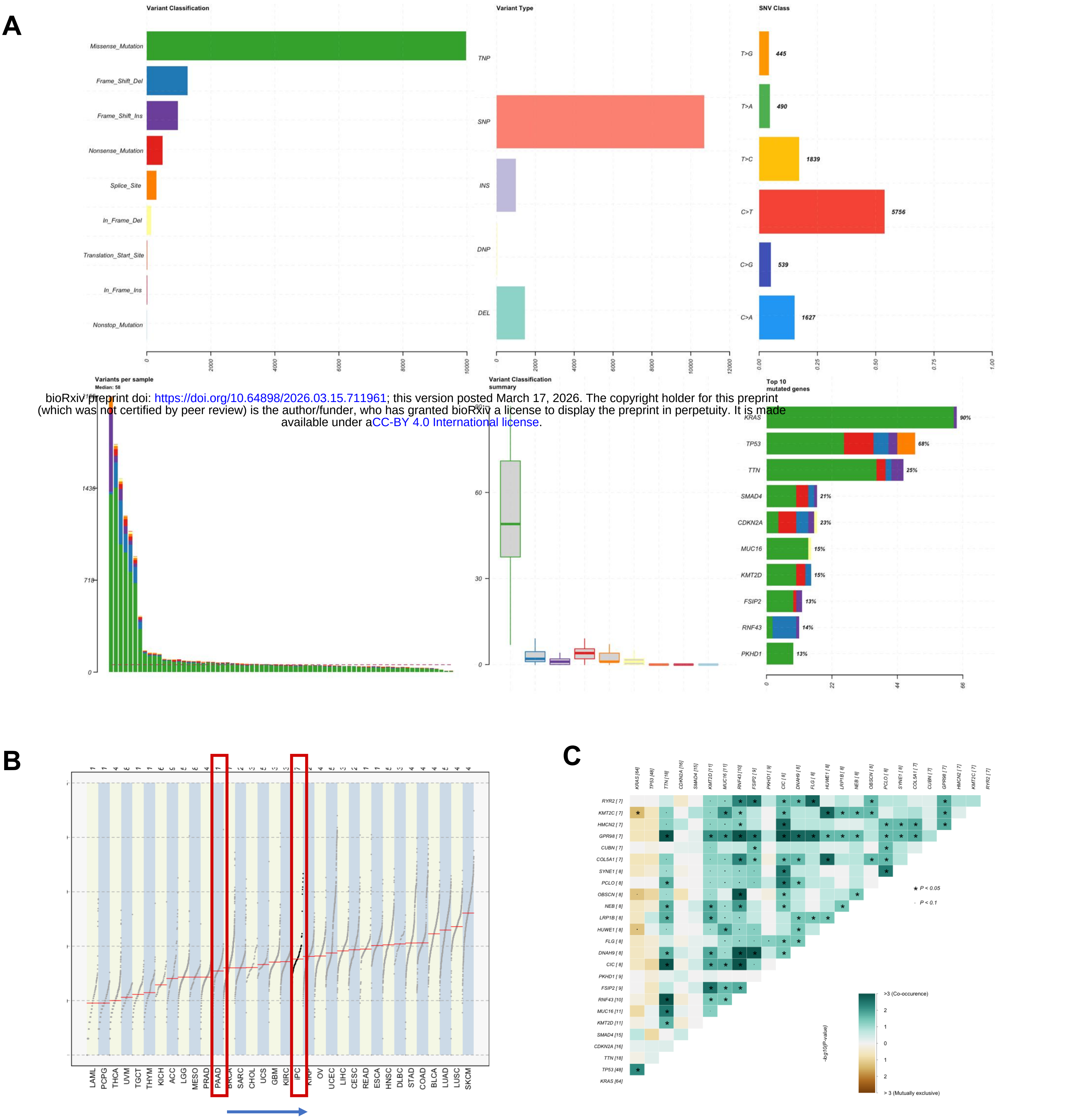

Figure S3. Somatic mutational landscape and frequent non-canonical mutations in DNA repair deficient subgroups

(A) Mutational summary of the iPC discovery cohort (N = 73) profiled by whole-exome sequencing. Panels include: **Top left**: Variant classification by type (missense, frameshift, etc.) **Top middle**: Variant type distribution (SNP, INS, DEL) **Top right**: Single-nucleotide variant (SNV) context, dominated by C>T transitions, **Bottom left**: Per-sample mutation count ranked by burden, **Bottom middle**: TMB distribution across samples, **Bottom right**: Most frequently mutated genes across the cohort, with color-coded subgroup contributions **(B)** Tumor mutation burden across tumor types from TCGA, highlighting immunogenic tumors (iPC) with higher median TMB compared to the general representative PDAC (PAAD). **(C)** Co-occurrence matrix of somatic mutations in frequently altered DDR and immune-relevant genes, revealing strong co-mutation patterns beyond canonical DDR genes (e.g., ARID1A, TP53, ZFHX3).

A

Characteristic	N	**dMMR**, N = 7[1]	**HRD**, N = 18[1]	**ncHRD**, N = 7[1]	**HRP**, N = 39[1]	**p-value**[2]
Age	71	75 (57 – 78)	63 (52 – 67)	66 (55 – 75)	66 (61 – 76)	0.37
Sex	71					0.072
F		3 (43)	12 (67)	2 (29)	12 (31)	
M		4 (57)	6 (33)	5 (71)	27 (69)	
Initial_Stage	71					<0.001
1		1 (14)	0 (0)	0 (0)	0 (0)	
2		1 (14)	3 (17)	0 (0)	0 (0)	
3		3 (43)	0 (0)	2 (29)	4 (10)	
4		2 (29)	15 (83)	5 (71)	35 (90)	
Tumor_Location	71					0.33
Body		2 (29)	1 (5.6)	1 (14)	11 (28)	
Head		3 (43)	6 (33)	4 (57)	12 (31)	
Others_overlap		1 (14)	8 (44)	1 (14)	6 (15)	
Tail		1 (14)	3 (17)	1 (14)	10 (26)	
Sample_Origin	71					0.008
Liver		1 (14)	10 (56)	4 (57)	28 (72)	
Lung		0 (0)	0 (0)	0 (0)	2 (5.1)	
Lymph_node		0 (0)	1 (5.6)	0 (0)	0 (0)	
Ovary		0 (0)	0 (0)	0 (0)	1 (2.6)	
Pancreas		5 (71)	6 (33)	2 (29)	2 (5.1)	
Peritoneum		1 (14)	1 (5.6)	1 (14)	6 (15)	

[1] Median (IQR); n (%)
[2] Kruskal-Wallis rank sum test; Fisher's exact test

bioRxiv preprint doi: https://doi.org/10.64898/2026.03.15.711961; this version posted March 17, 2026. The copyright holder for this preprint (which was not certified by peer review) is the author/funder, who has granted bioRxiv a license to display the preprint in perpetuity. It is made available under aCC-BY 4.0 International license.

B

Characteristic	N	**dMMR**, N = 7[1]	**HRD**, N = 18[1]	**ncHRD**, N = 7[1]	**HRP**, N = 39[1]	**p-value**[2]
KRAS	71					0.075
G12C		1 (14)	0 (0)	0 (0)	0 (0)	
G12D		3 (43)	8 (44)	4 (57)	22 (56)	
G12R		0 (0)	1 (5.6)	0 (0)	5 (13)	
G12V		0 (0)	8 (44)	1 (14)	8 (21)	
G13D		0 (0)	0 (0)	0 (0)	1 (2.6)	
I171Nfs*14		1 (14)	0 (0)	0 (0)	0 (0)	
Q61H		0 (0)	0 (0)	0 (0)	1 (2.6)	
Q61R		0 (0)	0 (0)	1 (14)	1 (2.6)	
WT		2 (29)	1 (5.6)	1 (14)	1 (2.6)	
First_Line_Platinum	71	3 (43)	16 (89)	4 (57)	22 (56)	0.044
OS	71	74 (26 – 105)	26 (12 – 36)	7 (7 – 25)	15 (9 – 24)	0.018
Ploidy	71	2.00 (2.00 – 2.15)	1.95 (1.83 – 2.00)	2.00 (1.85 – 2.60)	2.00 (1.90 – 2.80)	0.18
ZygosityHRD	25					0.11
Biallelic		0 (NA)	16 (89)	4 (57)	0 (NA)	
Monoallelic		0 (NA)	2 (11)	3 (43)	0 (NA)	
Unknown		7	0	0	39	
MSI_score	71	[illegible]	0.5 (0.2 – 0.9)	0.4 (0.2 – 1.9)	0.7 (0.4 – 1.2)	<0.001
IMPACT_HRD_score	67	5 (4 – 7)	55 (35 – 58)	27 (23 – 29)	23 (14 – 30)	<0.001
Unknown		1	1	1	1	
WES_TMB	71	35 (30 – 47)	3 (2 – 3)	1 (1 – 1)	1 (1 – 2)	<0.001
MDF_4Q	71					<0.001
1		0 (0)	0 (0)	3 (43)	14 (36)	
2		7 (100)	1 (5.6)	1 (14)	9 (23)	
3		0 (0)	2 (11)	3 (43)	13 (33)	
4		0 (0)	15 (83)	0 (0)	3 (7.7)	
Neoantigen_Burden	71	3,566 (2,749 – 4,544)	225 (172 – 356)	109 (94 – 144)	131 (97 – 173)	<0.001

[1] n (%); Median (IQR)
[2] Fisher's exact test; Kruskal-Wallis rank sum test

Table S1. Clinical, molecular and survival characteristics of immunogenic pancreatic cancer stratified by DNA repair deficient subgroups

(A–B) Clinical and genomic features across WES-profiled patients (N = 71) stratified by DNA repair status: mismatch repair–deficient (dMMR), homologous recombination–deficient (HRD), non-core HRD (ncHRD), and homologous recombination–proficient (HRP).
(A) Demographic and clinicopathologic characteristics including age, sex, stage, tumor location, and sample origin.
(B) Molecular metrics including ploidy, whole-genome duplication (WGD), MSIsensor score, zygosity, HRD score (IMPACT-HRD score), tumor mutational burden (TMB), and MMEJ Deletion Footprint (MDF) quartile.

Abbreviations: PC, pancreatic cancer; dMMR, mismatch repair deficient; HRD, homologous recombination deficient; ncHRD, non-core HRD; HRP, homologous recombination proficient; TMB, tumor mutation burden; FGA, fraction genome altered; ICB, immune checkpoint blockade; NK, natural killer; MMEJ, microhomology-mediated end joining; MDF, MMEJ Deletion Footprint.

A

Characteristic	N	HRD, N = 18[1]	HRP, N = 39[1]	p-value[2]
Age	57	64 (56 – 72)	66 (61 – 76)	0.12
Sex	57			0.030
F		11 (61)	12 (31)	
M		7 (39)	27 (69)	
Initial_Stage	57			0.027
2		2 (11)	0 (0)	
3		3 (17)	2 (5.1)	
4		13 (72)	37 (95)	
Tumor_Location	57			0.25
Body		5 (28)	13 (33)	
Head		9 (50)	11 (28)	
Tail		4 (22)	15 (38)	
OS	57	61 (22 – 91)	14 (7 – 25)	<0.001

[1] Median (IQR); n (%)
[2] Wilcoxon rank sum test; Pearson's Chi-squared test; Fisher's exact test

bioRxiv preprint doi: https://doi.org/10.64898/2026.03.15.711961; this version posted March 17, 2026. The copyright holder for this preprint (which was not certified by peer review) is the author/funder, who has granted bioRxiv a license to display the preprint in perpetuity. It is made available under aCC-BY 4.0 International license.

B

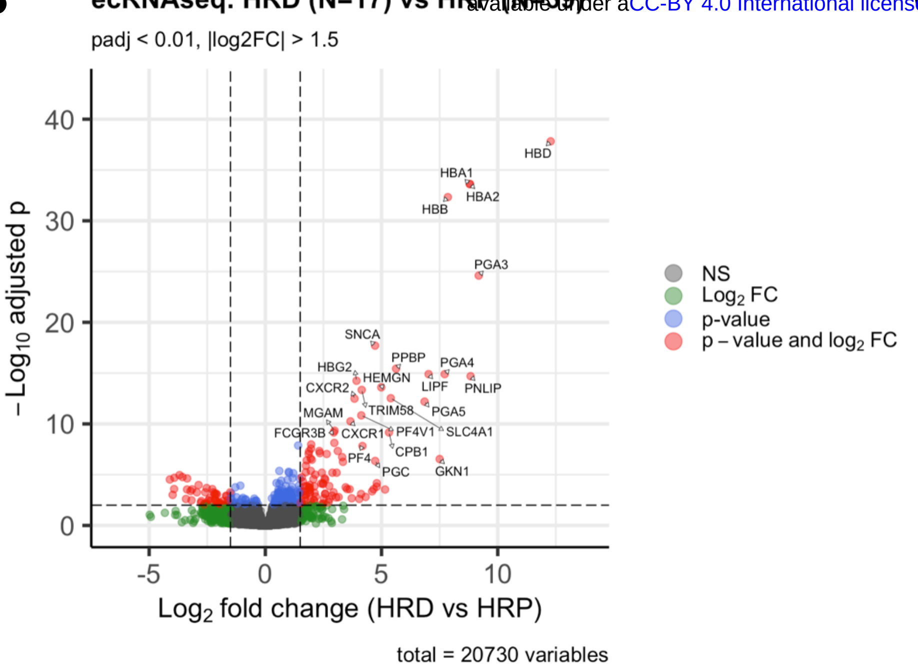

C

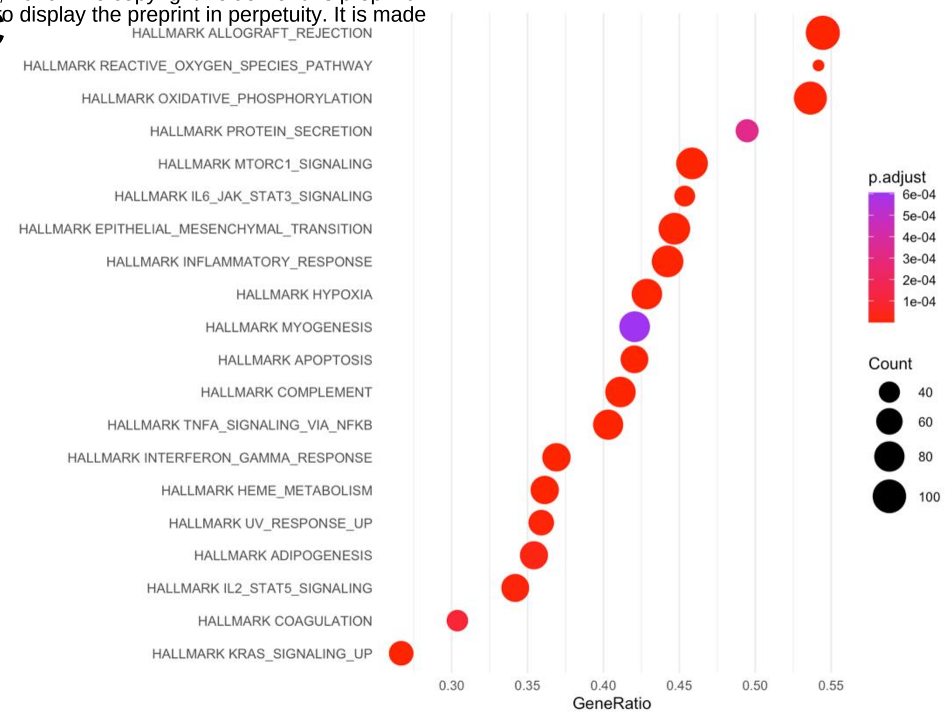

D

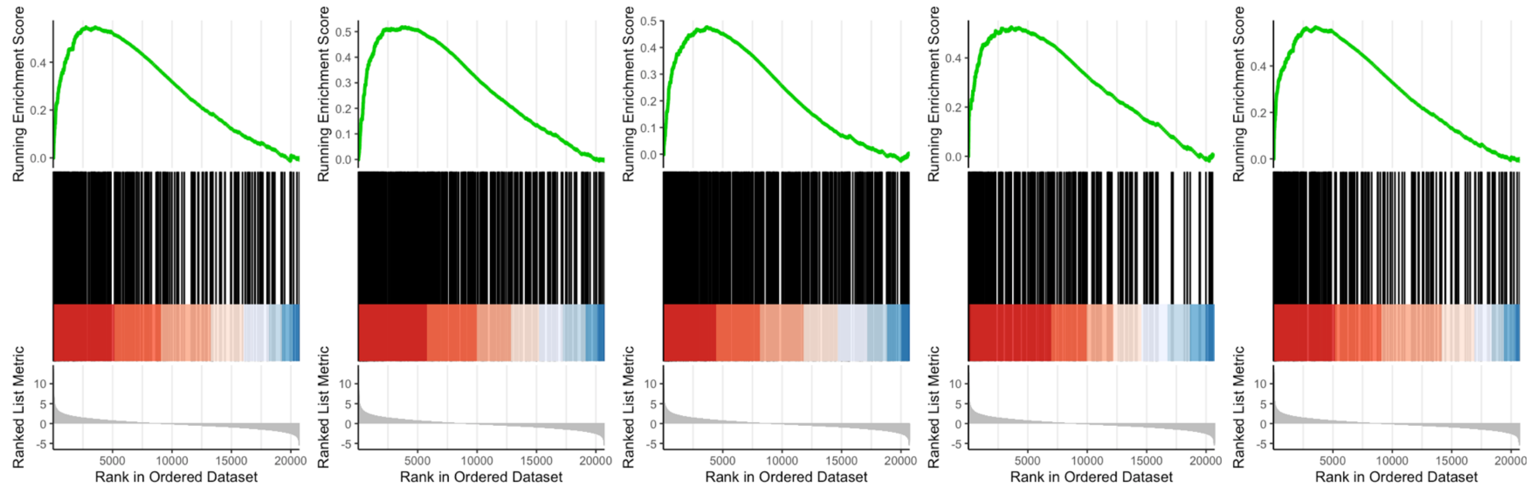

Description	Activation of immune response	Adaptive immune response	T cell activation	Humoral immune response	Immune response-regulating cell surface receptor signal
Gene Ontology	0002253	0002250	0042110	0006959	0002768
Enrichment score	0.55	0.52	0.48	0.53	0.57
Adjusted p-value	3.5e-22	7.0e-27	4.9e-24	3.1e-14	1.6e-21

Figure S4. Transcriptomic analysis reveals immunogenicity of homologous recombination deficient pancreatic cancer (HRD PC)

(**A**) Demographic and clinical variables including age, sex, stage, tumor location, and overall survival of the ecRNAseq dataset. **(B)** Volcano plot comparing bulk RNA-seq profiles between HRD (N=17) and HRP (N=39) tumors. Differential expression analysis reveals multiple upregulated immune genes in HRD tumors. Threshold: log2 fold-change >1.5 and adjusted p-value<0.01. See STAR Methods: Differential expression analysis. **(C)** Hallmark pathway enrichment analysis (GSEA) identifies immune-related pathways significantly enriched in HRD tumors. See STAR Methods: Hallmark gene set enrichment. **(D)** Gene set enrichment plots for key immune-related GO terms, with associated enrichment scores and adjusted p-values. Top enriched terms include activation of immune response, Adaptive immune response, T cell activation, humoral immune response and immune response-regulating cell surface receptor signal.

bioRxiv preprint doi: https://doi.org/10.64898/2026.03.15.711996; this version posted [illegible] 17, 2026. The copyright holder for this preprint (which was not certified by peer review) is the author/funder, who has granted bioRxiv a license to display the preprint in perpetuity. It is made available under aCC-BY 4.0 International license.

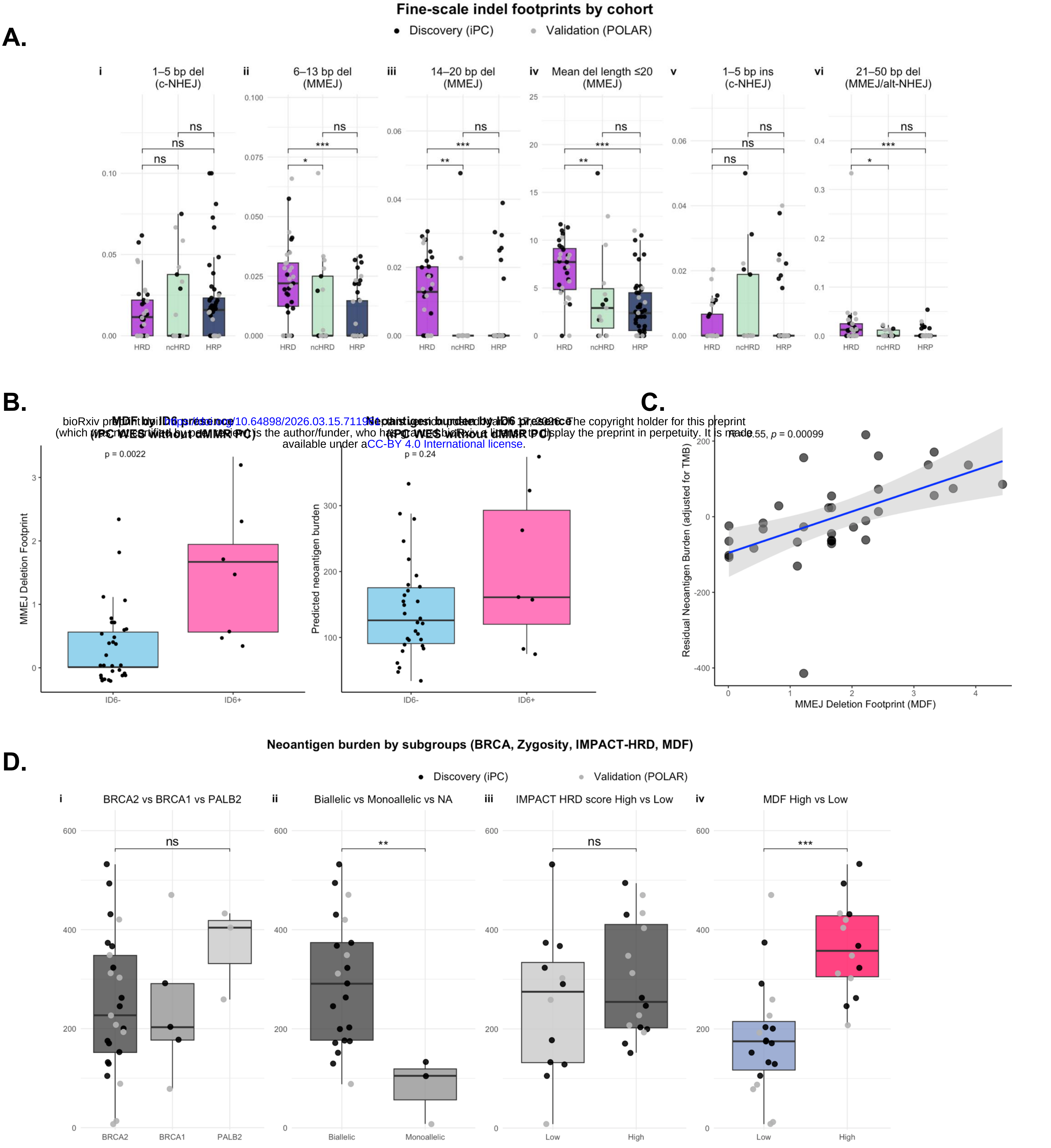

Figure S5. Genomic mutational patterns associated with POLQ-mediated MMEJ activity and neoantigen burden in HRD pancreatic cancer.

(A) Fine-scale indel footprint analysis used to derive the MMEJ Deletion Footprint (MDF), which quantifies deletion patterns characteristic of POLQ-mediated microhomology-mediated end joining (MMEJ). HRD tumors were enriched for deletions in the 6–20 bp range. Discovery iPC cohort samples are shown as black dots and validation POLAR cohort samples as grey dots. **p-values from Wilcoxon tests; **p < 0.01, *p < 0.001. (B) COSMIC indel signature ID6, a marker of MMEJ repair, was associated with higher MDF scores (p = 0.0022) but not with neoantigen burden (p = 0.24). (C) MDF remained positively associated with neoantigen burden after adjustment for tumor mutational burden (TMB) (Pearson R = 0.55, p = 0.00099). (D) Neoantigen burden across HRD-related genomic stratifications. Neoantigen burden was associated with HRD gene zygosity and MDF score but not with BRCA genotype or IMPACT-HRD score.

A

Characteristic	N	HRD, N = 18[1]	HRP, N = 39[1]	p-value[2]
KRAS	50			0.47
G12D		3 (27)	19 (49)	
G12R		3 (27)	7 (18)	
G12V		3 (27)	10 (26)	
Q61H		1 (9.1)	1 (2.6)	
WT		1 (9.1)	2 (5.1)	
Unknown		7	0	
IMPACT_HRD_score	39	57 (36 – 58)	19 (14 – 25)	<0.001
Unknown		9	9	
Plastic_state	56			>0.99
basal-like		2 (11)	6 (16)	
classical		16 (89)	32 (84)	
Unknown		0	1	

[1] n (%); Median (IQR)

[2] Fisher's exact test; Wilcoxon rank sum test

bioRxiv preprint doi: https://doi.org/10.64898/2026.03.15.711961; this version posted March 17, 2026. The copyright holder for this preprint (which was not certified by peer review) is the author/funder, who has granted bioRxiv a license to display the preprint in perpetuity. It is made available under aCC-BY 4.0 International license.

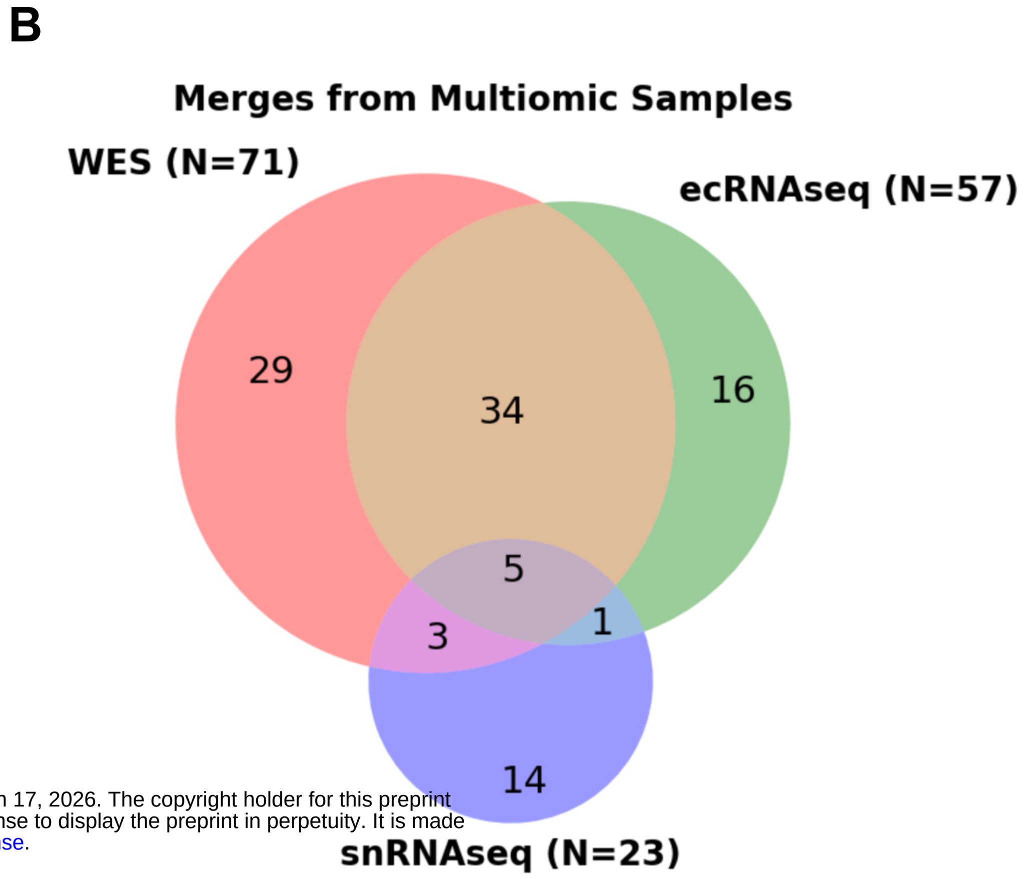

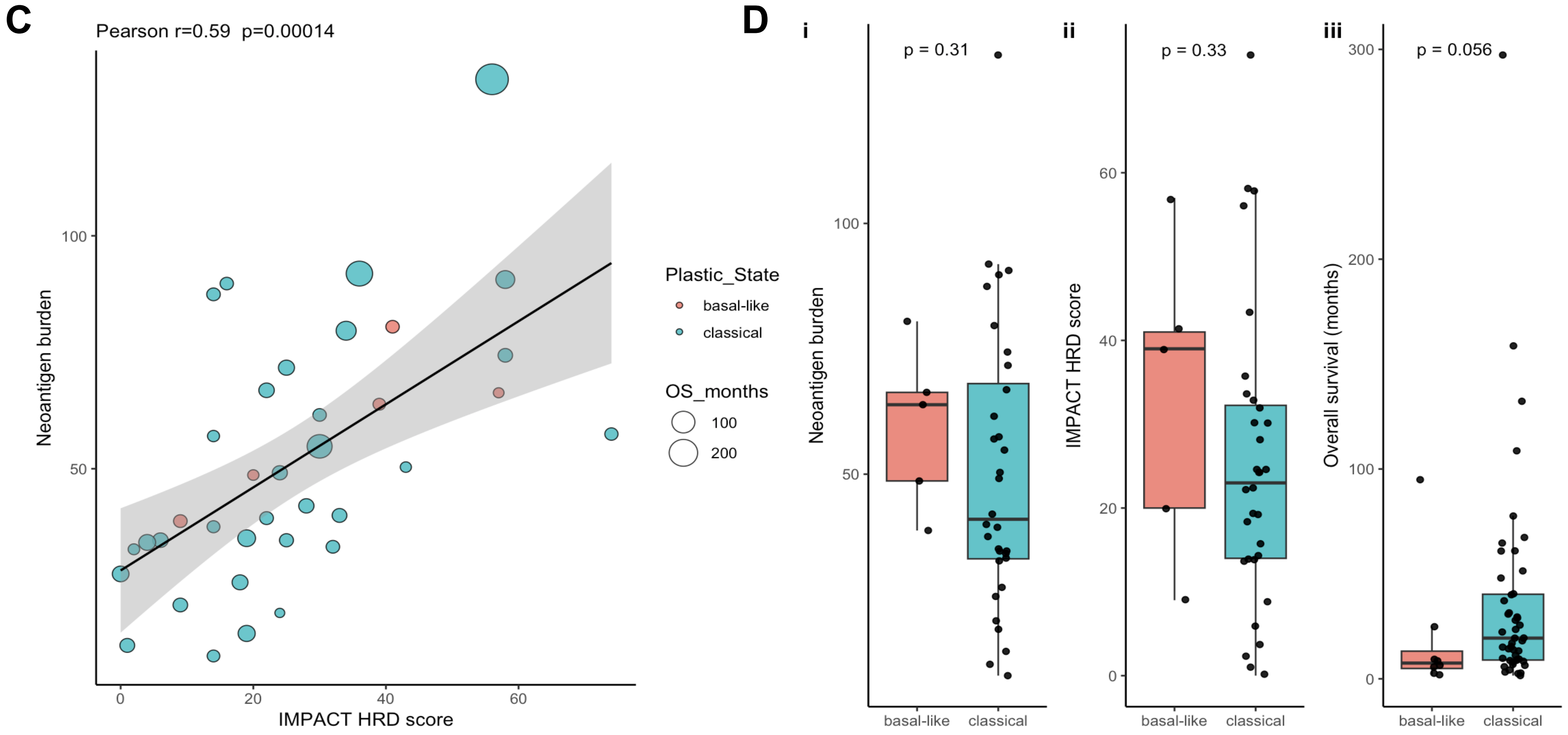

Figure S6. Plastic subtype classification is independent of genomic features of HRD PC

Transcriptional plastic subtype features comparison with clinic-genomic feature: **(A)** KRAS status, IMPACT-HRD score, Plastic state in PurIST available samples (N=56) by HRD and HRP **(B)** Venn Diagram for Merges from multiomic samples **(C)** Neoantigen Burden is associated with HRD score by IMPACT-HRD (R=0.59, p=0.00014). **(D)** Boxplots comparing basal-like and classical subtypes in by: (i) Neoantigen burden (p=0.31), (ii) IMPACT HRD score (p=0.33) and (iii) Overall survival (p=0.056). Classical tumors show longer survival despite comparable HRD score and neoantigen burdens, suggesting that plasticity state adds prognostic value.

A.

Characteristic	N	HRD, N = 17[1]	HRP, N = 6[1]	p-value[2]
age_at_dx	23	65 (57 – 67)	62 (59 – 64)	0.29
sex	23			0.37
F		10 (59)	2 (33)	
M		7 (41)	4 (67)	
race	23			0.39
Asian		1 (5.9)	2 (33)	
Black		1 (5.9)	0 (0)	
White		15 (88)	4 (67)	
stage_at_dx	23			0.22
2		5 (29)	0 (0)	
3		3 (18)	0 (0)	
4		9 (53)	6 (100)	
Bx_location	23			0.058
Liver		8 (47)	4 (67)	
Lung		0 (0)	1 (17)	
Lymph Node		0 (0)	1 (17)	
Pancreas		7 (41)	0 (0)	
Peritoneum		2 (12)	0 (0)	
surgery	23			0.42
0		2 (12)	0 (0)	
1		4 (24)	0 (0)	
2		11 (65)	6 (100)	
Firstline_plat	23	17 (100)	6 (100)	
Firstline_regimen	23			<0.001
FOLFIRINOX		0 (0)	3 (50)	
Gemcitabine/ Cisplatin		4 (24)	0 (0)	
Gemcitabine/ Cisplatin/ Veliparib		13 (76)	0 (0)	
Gemcitabine/ Nab-paclitaxel		0 (0)	3 (50)	
OS	23	19 (14 – 35)	16 (11 – 21)	0.25
OS_event	23	16 (94)	6 (100)	>0.99
LTS_V_STS	23			0.37
LTS		10 (59)	2 (33)	
STS		7 (41)	4 (67)	

[1] Median (IQR); n (%)
[2] Wilcoxon rank sum test; Fisher's exact test

bioRxiv preprint doi: https://doi.org/10.64898/2026.03.15.711961; this version posted March 17, 2026. The copyright holder for this preprint (which was not certified by peer review) is the author/funder, who has granted bioRxiv a license to display the preprint in perpetuity. It is made available under aCC-BY 4.0 International license.

B.

Characteristic	N	HRD, N = 17[1]	HRP, N = 6[1]	p-value[2]
HRD	23			<0.001
BRCA1		3 (18)	0 (0)	
BRCA2		14 (82)	0 (0)	
No_HRD		0 (0)	6 (100)	
cells	23	2,586 (2,026 – 3,534)	3,663 (1,470 – 4,966)	0.55
genes	23	16,007 (11,439 – 18,803)	18,078 (15,003 – 18,931)	0.51
WES	23			0.28
		1 (5.9)	0 (0)	
N		7 (41)	5 (83)	
Y		9 (53)	1 (17)	
Matched	23			0.019
N		7 (41)	6 (100)	
Y		10 (59)	0 (0)	
Neoantigen_available	23			0.37
N		10 (59)	5 (83)	
Y		7 (41)	1 (17)	
Plastic_State	23			0.65
		13 (76)	6 (100)	
Basal-like		1 (5.9)	0 (0)	
Classical		3 (18)	0 (0)	
IMPACT_HRD_available	23			0.14
N		11 (65)	6 (100)	
Y		6 (35)	0 (0)	
PDF_available	23			0.37
N		10 (59)	5 (83)	
Y		7 (41)	1 (17)	
ecRNAseq	23			0.058
N		9 (53)	6 (100)	
Y		8 (47)	0 (0)	
Bx_Timing	23			0.12
Baseline		10 (59)	6 (100)	
FU		7 (41)	0 (0)	

[1] n (%); Median (IQR)
[2] Fisher's exact test; Wilcoxon rank sum test

C.

Characteristic	N	BRCA1, N = 3[1]	BRCA2, N = 14[1]	No_HRD, N = 6[1]	p-value[2]
HRD_HRP	23				<0.001
HRD		3 (100)	14 (100)	0 (0)	
HRP		0 (0)	0 (0)	6 (100)	
LTS_V_STS	23				0.40
LTS		1 (33)	9 (64)	2 (33)	
STS		2 (67)	5 (36)	4 (67)	
OS	23	14 (14 – 22)	22 (14 – 36)	16 (11 – 21)	0.40
MHC_binding	8				0.036
Strong		0 (0)	6 (100)	0 (0)	
Weak		1 (100)	0 (0)	1 (100)	
Unknown		2	8	5	

[1] n (%); Median (IQR)
[2] Fisher's exact test; Kruskal-Wallis rank sum test

Table S2. Clinical and Molecular Characteristics of HRD and HRP Pancreatic Tumor Biopsies in the snRNA-seq Cohort

Table S2. Clinical and Molecular Characteristics of HRD and HRP Pancreatic Tumor Biopsies in the snRNA-seq Cohort

(A–B) Baseline demographics, clinical features, and genomic data from the 23 tumors profiled by single-nucleus RNA-seq (HRD=17, HRP=6). **(A)** No significant differences were observed in age, sex, race, tumor stage, biopsy site, or survival outcomes between HRD and HRP tumors. There were more liver biopsy samples HRD tumors were significantly enriched for *BRCA2* mutations and more likely to have received gemcitabine/cisplatin/veliparib as first-line therapy ($p<0.001$). **(B)** Molecular characteristics showed that HRD tumors more frequently had matched WES data and available neoantigen calls, with a trend toward greater MHC-I binding strength in predicted epitopes (not statistically significant). No differences were observed in cell or gene counts or plasticity state classification. **(C)** Stratified analysis comparing *BRCA1* mutation (N=3), *BRCA2* mutation (N=14), and non-HRD (N=6) tumors.

bioRxiv preprint doi: https://doi.org/10.64898/2026.03.15.711961; this version posted March 17, 2026. The copyright holder for this preprint (which was not certified by peer review) is the author/funder, who has granted bioRxiv a license to display the preprint in perpetuity. It is made available under aCC-BY 4.0 International license.

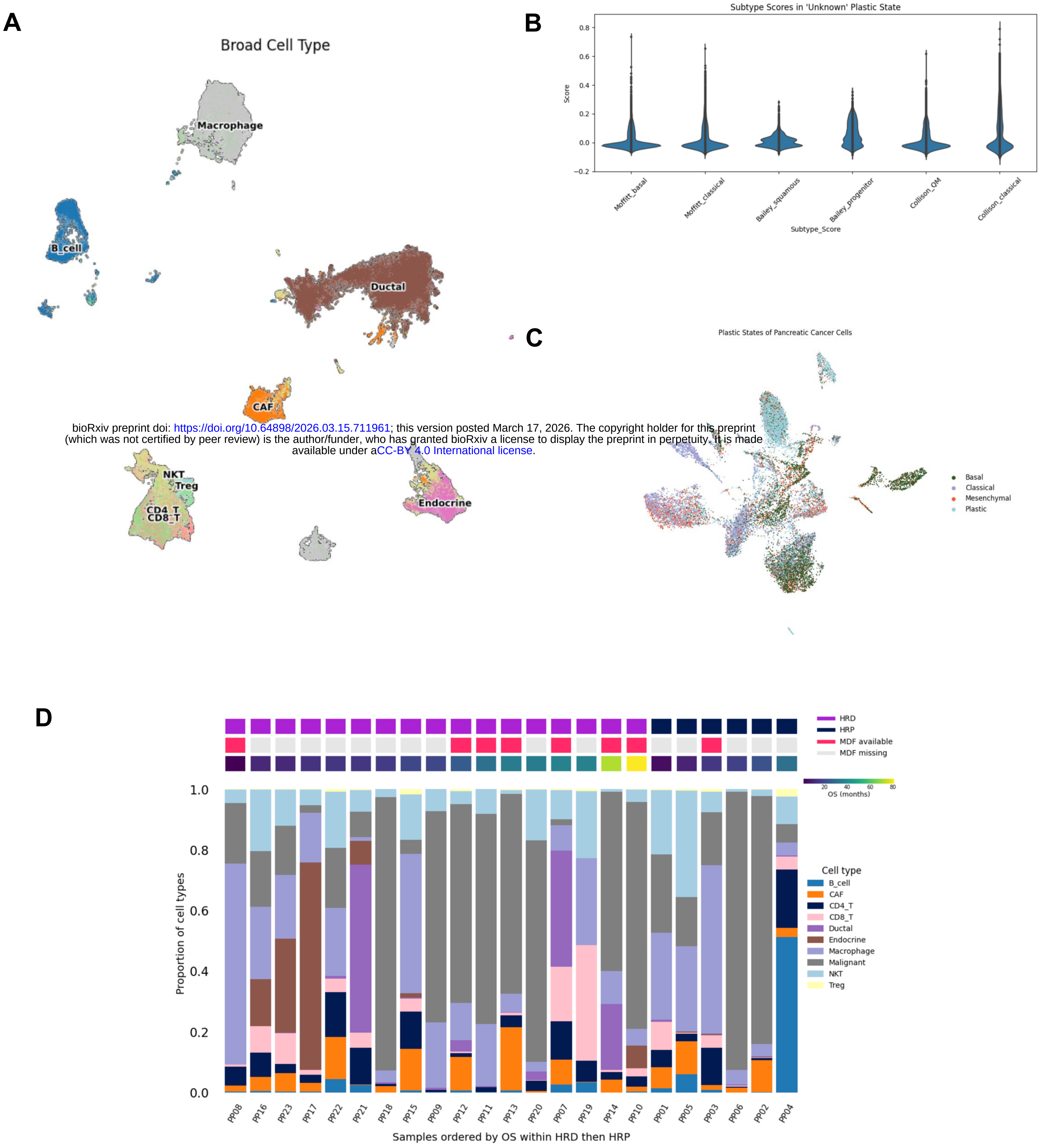

bioRxiv preprint doi: https://doi.org/10.64898/2026.03.15.711961; this version posted March 17, 2026. The copyright holder for this preprint (which was not certified by peer review) is the author/funder, who has granted bioRxiv a license to display the preprint in perpetuity. It is made available under aCC-BY 4.0 International license.

Figure S7. Single nucleus transcriptomics reveals malignant cell plastic states concordant with established pancreatic cancer subtypes.

Single-nucleus RNA-seq delineates transcriptional plasticity in pancreatic cancer malignant cells: **(A)** broad cell type annotation across tumor biopsies (N=23), (**B)** malignant cell plastic subtype scoring using gne signatures corresponding to classical, basal-like, progenitor, and quasi-mesenchymal programs, **(C)** UMAP visualization of malignant cell plastic states **(D)** proportional composition of cell types per sample in the increasing order of overall survival and HRD status with annotation bars and MDF availability

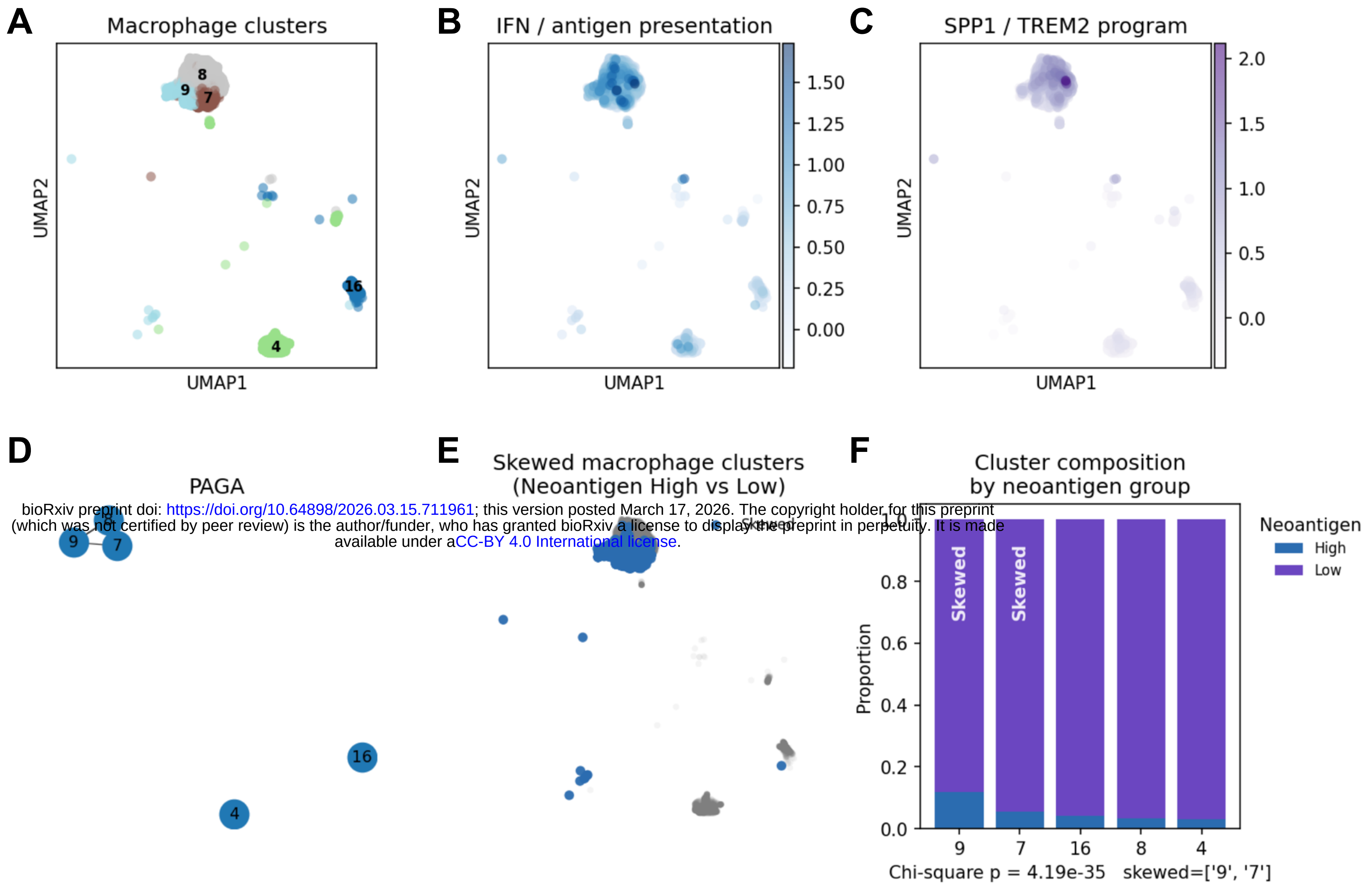

bioRxiv preprint doi: https://doi.org/10.64898/2026.03.15.711961; this version posted March 17, 2026. The copyright holder for this preprint (which was not certified by peer review) is the author/funder, who has granted bioRxiv a license to display the preprint in perpetuity. It is made available under aCC-BY 4.0 International license.

G

<table>
<tr><th></th><th>Program</th><th>Side</th><th>Intended_cells</th><th>Genes_requested</th><th>Genes_used_present</th><th>n_genes_used</th></tr>
<tr><td>0</td><td>Cytolytic Effector</td><td>Receptor-side (T cells)</td><td>CD4_T, CD8_T, Treg</td><td>PRF1, GZMB, GNLY, NKG7, EOMES</td><td>PRF1, GZMB, GNLY, NKG7, EOMES</td><td>5</td></tr>
<tr><td>1</td><td>IFN_r Response</td><td>Receptor-side (T cells)</td><td>CD4_T, CD8_T, Treg</td><td>IFNG, CXCL10, ISG15, IFIT3, HLA-DRA, CD74</td><td>IFNG, CXCL10, ISG15, IFIT3, HLA-DRA, CD74</td><td>6</td></tr>
<tr><td>2</td><td>Dysfunction</td><td>Receptor-side (T cells)</td><td>CD4_T, CD8_T, Treg</td><td>TOX, HAVCR2, ENTPD1, TIGIT, LAG3, PDCD1</td><td>TOX, HAVCR2, ENTPD1, TIGIT, LAG3, PDCD1</td><td>6</td></tr>
<tr><td>3</td><td>Treg Program</td><td>Receptor-side (T cells)</td><td>CD4_T, CD8_T, Treg</td><td>FOXP3, IL2RA, CTLA4, IKZF2, TNFRSF18</td><td>FOXP3, IL2RA, CTLA4, IKZF2, TNFRSF18</td><td>5</td></tr>
<tr><td>4</td><td>PD-L1|CD274</td><td>Ligand/APC availability</td><td>Macrophage, Mac_MHCIIhi, Malignant</td><td>CD274</td><td>CD274</td><td>1</td></tr>
<tr><td>5</td><td>Costim (CD80/86)</td><td>Ligand/APC availability</td><td>Macrophage, Mac_MHCIIhi, Malignant</td><td>CD80, CD86</td><td>CD80, CD86</td><td>2</td></tr>
<tr><td>6</td><td>MHC_II|APC</td><td>Ligand/APC availability</td><td>Macrophage, Mac_MHCIIhi, Malignant</td><td>HLA-DRA, HLA-DRB1, CD74</td><td>HLA-DRA, HLA-DRB1, CD74</td><td>3</td></tr>
<tr><td>7</td><td>CD40L</td><td>Ligand/APC availability</td><td>Macrophage, Mac_MHCIIhi, Malignant</td><td>CD40LG</td><td>CD40LG</td><td>1</td></tr>
<tr><td>8</td><td>STING|IFN_ab</td><td>Ligand/APC availability</td><td>Macrophage, Mac_MHCIIhi, Malignant</td><td>IFIT1, IFIT2, IFIT3, ISG15, MX1, OAS1, OAS2, D...</td><td>IFIT1, IFIT2, IFIT3, ISG15, MX1, OAS1, OAS2, D...</td><td>10</td></tr>
</table>

Figure S8. Macrophage clustering, trajectory structure, and immune synapse gene programs.

(A) UMAP of macrophage clusters identified from the myeloid compartment.
(B) Module score for interferon and antigen presentation programs across macrophage populations.
(C) Module score for suppressive TAM programs defined by **SPP1** and **TREM2**.
(D) PAGA graph showing connectivity among macrophage clusters, illustrating relationships among macrophage states.
(E) UMAP highlighting macrophage clusters skewed by tumor neoantigen burden (high vs low).
(F) Cluster composition by neoantigen group (chi-square test shown).
(G) Gene programs used to quantify immune synapse signaling, including cytolytic T cell, interferon response, dysfunction/Treg programs, and APC-associated ligand programs (PD-L1, CD80/CD86, MHC-II, CD40L, STING/IFN).
